# Transplantation of human oligodendrocyte progenitor cells in an animal model of diffuse traumatic axonal injury: survival and differentiation

**DOI:** 10.1186/s13287-015-0087-0

**Published:** 2015-05-14

**Authors:** Leyan Xu, Jiwon Ryu, Hakim Hiel, Adarsh Menon, Ayushi Aggarwal, Elizabeth Rha, Vasiliki Mahairaki, Brian J Cummings, Vassilis E Koliatsos

**Affiliations:** Division of Neuropathology, Department of Pathology, The Johns Hopkins University School of Medicine, Baltimore, MD 21205 USA; Department of Otolaryngology-Head and Neck Surgery, The Johns Hopkins University School of Medicine, Baltimore, MD 21205 USA; Departments of Physical and Medical Rehabilitation, Neurological Surgery, and Anatomy and Neurobiology, Sue and Bill Gross Stem Cell Research Center, Institute for Memory Impairments and Neurological Disorders, University of California at Irvine, Irvine, CA 92697 USA; Department of Neurology, The Johns Hopkins University School of Medicine, Baltimore, MD 21205 USA; Department of Psychiatry and Behavioral Sciences, The Johns Hopkins University School of Medicine, Baltimore, MD 21205 USA

## Abstract

**Introduction:**

Diffuse axonal injury is an extremely common type of traumatic brain injury encountered in motor vehicle crashes, sports injuries, and in combat. Although many cases of diffuse axonal injury result in chronic disability, there are no current treatments for this condition. Its basic lesion, traumatic axonal injury, has been aggressively modeled in primate and rodent animal models. The inexorable axonal and perikaryal degeneration and dysmyelination often encountered in traumatic axonal injury calls for regenerative therapies, including therapies based on stem cells and precursors. Here we explore the proof of concept that treatments based on transplants of human oligodendrocyte progenitor cells can replace or remodel myelin and, eventually, contribute to axonal regeneration in traumatic axonal injury.

**Methods:**

We derived human oligodendrocyte progenitor cells from the human embryonic stem cell line H9, purified and characterized them. We then transplanted these human oligodendrocyte progenitor cells into the deep sensorimotor cortex next to the corpus callosum of nude rats subjected to traumatic axonal injury based on the impact acceleration model of Marmarou. We explored the time course and spatial distribution of differentiation and structural integration of these cells in rat forebrain.

**Results:**

At the time of transplantation, over 90 % of human oligodendrocyte progenitor cells expressed A2B5, PDGFR, NG2, O4, Olig2 and Sox10, a profile consistent with their progenitor or early oligodendrocyte status. After transplantation, these cells survived well and migrated massively via the corpus callosum in both injured and uninjured brains. Human oligodendrocyte progenitor cells displayed a striking preference for white matter tracts and were contained almost exclusively in the corpus callosum and external capsule, the striatopallidal striae, and cortical layer 6. Over 3 months, human oligodendrocyte progenitor cells progressively matured into myelin basic protein(+) and adenomatous polyposis coli protein(+) oligodendrocytes. The injured environment in the corpus callosum of impact acceleration subjects tended to favor maturation of human oligodendrocyte progenitor cells. Electron microscopy revealed that mature transplant-derived oligodendrocytes ensheathed host axons with spiral wraps intimately associated with myelin sheaths.

**Conclusions:**

Our findings suggest that, instead of differentiating locally, human oligodendrocyte progenitor cells migrate massively along white matter tracts and differentiate extensively into ensheathing oligodendrocytes. These features make them appealing candidates for cellular therapies of diffuse axonal injury aiming at myelin remodeling and axonal protection or regeneration.

**Electronic supplementary material:**

The online version of this article (doi:10.1186/s13287-015-0087-0) contains supplementary material, which is available to authorized users.

## Introduction

Axonal injury is the defining feature of diffuse axonal injury (DAI), but is also present in blast injuries [1], chronic traumatic encephalopathy [2], and even mild head injuries [3]. Axonal damage in models of DAI is referred to as traumatic axonal injury (TAI), a term often used interchangeably with DAI [4, 5]. In the case of DAI, axonal injury causes disconnection of neural circuits at multiple central nervous system (CNS) sites [6–8] and can lead to a number of neurological impairments, including long-term memory problems, emotional disturbances, unconsciousness, and/or a persistent vegetative state. These neurological impairments have no satisfactory treatment besides symptomatic alleviation of various subsyndromes with physical, occupational, speech and language therapy and various categories of CNS-acting drugs including antispasmodics, antidepressants, and mood stabilizers. Although some retraining of circuits is anticipated over time and syndromic pharmacotherapies have some effectiveness, most patients with DAI still remain severely symptomatic years and decades later.

Stem cell therapy presents a promising treatment approach for traumatic brain injury (TBI). Some early success in models of ischemic brain injury [9] has encouraged the use of stem cell or neural precursor (NP) transplantation, primarily in models of focal TBI [10]. Much less is known about the role of stem cell therapies in DAI/TAI. Axonal repair as a target of treatment separate from nerve cell regeneration is not as well established in TBI as in spinal cord injury, and this is especially true with the problem of myelin repair/remyelination [11]. However, demyelination appears to contribute to degeneration of axons in TAI [12, 13] and TAI is associated with active and ongoing attempts at axonal repair [14]. Therefore, adding exogenous oligodendrocyte progenitor cells (OPCs) may furnish competent oligodendrocytes that can assist in remyelination/myelin remodeling and prevent axonal degeneration or help myelinate regenerating axons in TAI.

Animal models are invaluable tools in establishing proof of concept that remyelination by exogenously provided oligodendrocytes is possible in TAI settings. Models of inertial acceleration and impact acceleration (IA) are frequently used for experimental studies of DAI/TAI [5, 15]. In the present study we use the IA model of DAI/TAI [16] and transplant human embryonic stem cell (ESC)-derived OPCs (hOPCs) into the deep sensorimotor cortex next to the corpus callosum. Our findings indicate that exogenous hOPCs differentiate into mature oligodendrocytes, migrate extensively along white matter tracts, and begin to myelinate host axons. Our data are consistent with the view that stem cell grafts may serve as effective myelin remodeling tools in TBI scenarios featured by DAI/TAI.

## Materials and methods

### Human embryonic stem cell culture and differentiation to human oligodendrocyte progenitor cells

The human ESC line H9 from WiCell (Madison, WI, USA) was maintained according to standard stem cell culture protocols. H9 cells (WA-09; passages 30 to 41) were grown on mitotically inactivated mouse embryonic fibroblasts essentially as described in [17]. hOPCs were generated through extensive passaging as neurospheres based on the method of Hu and colleagues [18, 19] with minor modifications (Additional file 1: Fig. S1). The ventralizing factor sonic hedgehog (SHH; 100 ng/mL) along with the caudalizing factor retinoic acid (0.1 μM) were used to initially pattern neuroepithelial cells; glial differentiation medium (GDM; Dulbecco's modified Eagle's medium (DMEM)/F12, B27 without vitamin A, N1, MEM-NEAA, cAMP, biotin, 60 ng/mL triiodothyronine, 10 ng/mL platelet-derived growth factor (PDGF)-AA, insulin-like growth factor (IGF)1 and neurotrophin (NT)3) was used for further differentiation. Cells were trypsinized with TrypLE (Life Technologies, Grand Island, NY, USA) at day 84 after induction of differentiation, counted, and plated on p-L-ornithine- and laminin-coated plates. Cells were grown in GDM supplemented with PDGF-AA, IGF1 and NT3 for 12 days, then trypsinized, counted, and resuspended at high concentration (2.0 × 10^8^ per mL), and finally transplanted on day 98 after induction of differentiation.

### Characterization of human oligodendrocyte progenitor cells used for transplantation with immunocytochemistry

Two weeks before transplantation (on day 84, a time point chosen to correspond to the remaining time in differentiation of hOPCs destined for transplantation), hOPC neurospheres were trypsinized with TrypLE and counted. Twenty thousand cells were plated on polyornithine- and laminin-coated coverslips or Matrigel-coated four-well slide chambers and cultured in GDM supplemented with PDGF, IGF and NT3 for 2 weeks. Cultures were then fixed with 4 % paraformaldehyde in phosphate-buffered saline for 20 minutes and then subjected to immunocytochemistry with the oligodendrocytic markers A2B5, platelet-derived growth factor receptor (PDGFR)α, NG2, Sox10, and O4; the neuronal marker type III-tubulin epitope J1 (TUJ1); astrocyte marker glial fibrillary acidic protein (GFAP); and the mesodermal marker bone morphogenetic protein 4 (Table 1).

**Table 1:** Primary antibodies used for immunocytochemistry, immunohistochemistry and ultrastructural immunohistochemistry

Target phenotypes	Target proteins/epitopes	Host	Dilution	Vendor
Human cell markers	Human Nuclei Protein antibody (HNu)	Mouse	1:1,000	Millipore, Billerica, MA, USA
	Human cytoplasm-specific antibody (SC121)	Mouse	1:3,000	StemCells, Inc., CA, USA
Neuron (*in vivo*)	Type III-tubulin epitope J1 (TUJ1)	Rabbit	1:400	Covance, Berkeley, CA, USA
Neuron (*in vitro*)	Type III-tubulin epitope J1 (TUJ1)	Mouse	1:100	Sigma, Saint Louis, MO, USA
Mitotic marker	Ki67 antigen (NCL-Ki67p)	Rabbit	1:400	Novocastra Labs, Newcastle, UK
Axon	Neurofilament heavy chain	Rabbit	1:200	Sigma, Saint Louis, MO, USA
Progenitor/early oligodendrocyte	Olig2	Goat	1:50	Santa Cruz, Santa Cruz, CA, USA
	Platelet-derived growth factor receptor α (PDGFRα)	Rabbit	1:100	Santa Cruz, Santa Cruz, CA, USA
	A2B5	Mouse	1:200	Millipore, Billerica, MA, USA
	NG2	Rabbit	1:100	Millipore, Billerica, MA, USA
	O4	Mouse	1:100	Millipore, Billerica, MA, USA
Late oligodendrocyte	Myelin basic protein (MBP)	Rabbit	1:200	Abcam, Cambridge, MA, USA
	Adenomatous polyposis coli protein (APC)	Rabbit	1:200	Novus Biologicals, Littleton, CO, USA
Astrocyte (*in vivo*)	Glial fibrillary acidic protein (GFAP)	Rabbit	1:400	Dako, Carpinteria, CA, USA
Astrocyte (*in vitro*)	Glial fibrillary acidic protein (GFAP)	Rabbit	1:100	Millipore, Billerica, MA, USA
Mesodermal marker	Bone morphogenetic protein-4 (BMP4)	Mouse	1:300	Millipore, Billerica, MA, USA

### Animals and surgical procedures

Ten-week old male nude rats (Crl:NIH-*Foxn1*^*rnu*^; Charles River, Wilmington, MA, USA) were used for hOPC transplantation. Nude rats were chosen because immunodeficient animals yield greater engraftment and survival of human cells than immunocompetent animals treated with immunosuppressants [20]. All surgical procedures were carried out according to protocols approved by the Animal Care and Use Committee of the Johns Hopkins Medical Institutions using gas anesthesia (isoflurane:oxygen:nitrous oxide = 1:33:66) and aseptic methods. In order to explore the fate of transplanted hOPCs and compare differentiation between injured and uninjured scenarios, animals were separated into IA and sham groups. In the IA group, animals were subjected to injury with full artificial ventilation as described by Marmarou and colleagues [16]. In the present experiments, we employed a severe TBI regimen using a 450 g weight that was freely dropped onto the steel disc through a Plexiglass tube from a height of 2 meters. In the sham group, animals received all aspects of the regimen except the injury itself (weight on the steel disc). One week after injury, a time point that appears to optimize survival and differentiation [21, 22], 200,000 live hOPCs were transplanted into two sites 1 mm apart in the right deep motor cortex next to the corpus callosum (1 mm and 0 mm anterior to bregma, 2 mm lateral to midline and 3 mm ventral to pia) of either injured (*n* = 10) or sham (*n* = 5) animals using procedures that have been detailed in our published work [21, 23, 24]. To explore the progress of differentiation of transplanted hOPCs in the TAI environment, animals in the TAI group were allowed to survive for 6 weeks and 3 months. Sham animals with transplanted hOPCs were euthanized at 3 months.

### Histology, immunohistochemistry and microscopy

Brain tissues were prepared from animals perfused transcardially with 4 % phosphate-buffered paraformaldehyde. The axonal injury, survival, location and phenotypic fate of hOPC grafts were assessed with ABC peroxidase immunohistochemistry (IHC) and dual-label fluorescent IHC in serial coronal or sagittal sections (40 μm) through the brain as described previously [22, 24, 25]. Axonal injury was studied with well-established TAI markers, including an antibody against the amyloid precursor protein (APP), the monoclonal antibody RMO14 binding to the rod domain of neurofilaments H and M, and a monoclonal antibody against the 68-kDa light chain neurofilament protein. hOPC survival was studied with human-specific nuclei (HNu) or human-specific cytoplasm (SC121) antibody using immunoperoxidase or immunofluorescence labeling. Differentiation was studied with dual-label immunofluorescence combining HNu or SC121 with other oligodendrocyte markers - that is, the progenitor and early marker PDGFRα, the early markers O4 and GalC and late markers myelin basic protein (MBP) and adenomatous polyposis coli protein (APC). Appositions between axons and transplant-derived oligodendrocytes were visualized with the combination of antibodies against the heavy neurofilament subunit (NF-H) and the SC121 epitope as generic axonal and transplant-derived cell markers, respectively. The nuclear mitotic marker Ki67, the early neuronal marker TUJ1 and the astroglial cell marker GFAP were also used in separate co-localization experiments with HNu or SC121 as described elsewhere [22, 24, 26, 27]. All antibody information is listed in Table 1. The Gallyas silver staining method [28] was used to evaluate injured and/or degenerating axons and terminals. For this purpose, sections were processed with a commercially available kit (NeuroSilver kit II; FD Neurotechnologies, Ellicott City, MD, USA) as described previously [29].

Stained sections were studied on a Zeiss Axiophot microscope equipped for epifluorescence (Diagnostic Instruments Inc., Sterling Heights, MI, USA) or a Zeiss LSM 510 inverted confocal microscope (Carl Zeiss Inc., Oberkochen, Germany). Confocal microscopic images were captured with pinhole set at 0.8 μm to ensure co-localization of multiple labels at the same resolution level as semithin sections. Three-dimensional reconstruction by Z-stack scanning through regions of interest was acquired with LSM software (Carl Zeiss Inc., Oberkochen, Germany). Adobe Photoshop 7.0 software (Adobe Systems, San Jose, CA, USA) was used for montaging and image processing. All staining, image collection, and quantification were done in a fashion blind to group assignment.

### Ultrastructural immunohistochemistry

Myelin formation by transplanted hOPCs was assessed ultrastructurally with electron microscopy using standard pre-embedding immunoperoxidase-3,3'-diaminobenzidine IHC for the human cytoplasmic antigen SC121 as described previously [23–25, 30]. Briefly, brain sections prepared as in the previous section were treated with a solution containing 4 % paraformaldehyde and 0.2 % glutaraldehyde for 24 hours. Sections were then rinsed in 0.1 M phosphate buffer (pH 7.3) for 3 to 10 minutes, immersed in 1 % osmium tetroxide for 15 minutes, dehydrated in graded concentrations of ethanol, embedded in Poly/Bed 812 (Polysciences Inc., Warrington, PA, USA), polymerized at 60 °C for 72 hours, and then finally embedded in BEEM® capsules (Electron Microscopy Sciences, Hatfield, PA, USA). Half the sections were stained en block in uranyl acetate prior to embedding. Serial ultrathin sections were collected on Formvar-coated slotted grids and viewed with a Hitachi H7600 transmission electron microscope equipped with a 2 k×2 k bottom mount AMT XR-100 CCD camera (Hitachi High-Technologies Corporation, Tokyo, Japan). Only sections that were not stained with uranyl acetate were used for studying ensheathment profiles originating in hOPC transplants.

### Stereological quantification of human oligodendrocyte progenitor cell survival and differentiation

Numbers of surviving hOPCs were counted in serial, systematically and randomly sampled coronal sections based on the optical fractionator concept with the aid of a motorized stage Axioplan microscope (Carl Zeiss Inc.) equipped with Stereo Investigator (MicroBrightField Bioscience, lliston, VT, USA) as described previously [22]. To evaluate the migration and possible final residing location of differentiating hOPCs, only the contralateral side of transplantation was examined. hOPCs in corpus callosum and cortex were also counted separately for this purpose. Every twelfth serial coronal section through the transplant/injury site was selected for stereological analysis. The counting frame was set at 50×50 μm and the sampling grid and counting depth were 200×200 μm and 10 μm, respectively. Cells around the transplantation site were not counted because of difficulties in discerning individual cells in the densely packed center of the transplant.

Differentiation of survived hOPCs was estimated in a non-stereological fashion as described previously [27]. Briefly, we counted the total number of SC121(+) cells, as well as cells dually labeled with SC121 and the mature oligodendrocyte marker MBP from our immunofluorescent preparations, on randomly selected fields of cortex and corpus callosum using 40× magnification and avoiding the transplantation site. At least three fields in each of four serial sections were used from each animal. Numbers of SC121(+) and double-labeled profiles were pooled from each case and grouped per experimental protocol. Average numbers of single- and double-labeled cells were generated for two TBI groups and one Sham group (*n* = 5 per group). Differentiation rate was expressed as percentage of SC121 and MBP double-labeled cells in the population of SC121(+) cells.

### Migration mapping of oligodendrocyte lineage cells derived from human oligodendrocyte progenitor cell grafts

The positions of all SC121(+) cells were mapped on every twelfth coronal section through brain levels containing the grafted cells and their lineage using Neurolucida software (MicroBrightField Bioscience). Representative cells differentiated from hOPCs and their processes were also traced with Neurolucida software.

### Statistical methods

Variance between and across samples of numbers of oligodendrocyte-lineage cells classified by experimental history (IA versus sham), migratory destination in brain (corpus callosum versus neocortex), and time point after transplantation (6 weeks or 3 months) was analyzed with two-way analysis of variance (ANOVA) or *t* test. In the case of ANOVA, significant differences were further analyzed with *post hoc* tests to reveal important main effects or interactions. Statistical analyses were performed with STATISTICA 8.0 (StatSoft Inc., Tulsa, OK, USA).

## Results

### Axonal injury in nude rats using the impact acceleration model

**Fig. 1:**
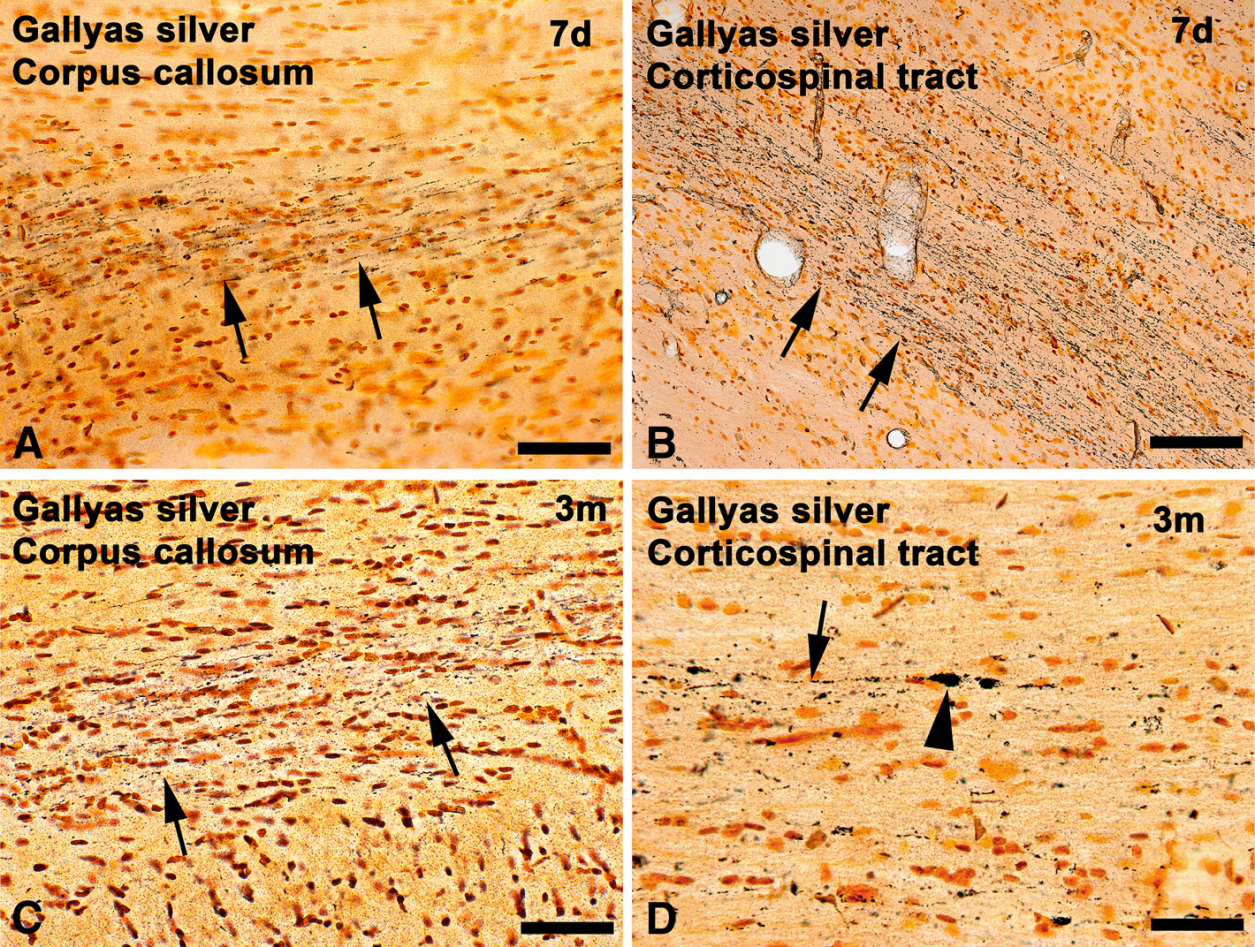
Traumatic axonal injury in impact acceleration-injured nude rats as demonstrated by Gallyas silver staining. One week after exposure of nude rats to severe impact acceleration injury, argyrophilic axonal degeneration is pronounced in (**a**) the corpus callosum and (**b**) the corticospinal tract (arrows). **c,d** At 3 months after injury, degenerating axons are still evident in these regions (arrows). Axonal bulbs are also present in (d) (arrowhead). Scale bars = 50 μm

Immunocompromised nude rats were used here to avoid immune rejection of human cell xenografts into rodent brain [20]. Because the original IA model was developed in Sprague-Dawley [16] and Wistar [31] rats, we first explored whether the same IA settings as the ones used in those strains can cause TAI in nude rats. Induction of TAI was studied with IHC strategies routinely used in TBI studies - that is, antibodies against APP, the monoclonal antibody RMO14 binding to the rod domain of neurofilament heavy and medium chains that are exposed after lesion-induced sidearm proteolysis, and a monoclonal antibody against the 68-kDa light chain neurofilament protein (NF68). IHC was used in brain sections from nude rats exposed to a standard severe IA injury (450 g weight drop from a height of 2 meters) [16]. Tissues were also processed with a modification of the Gallyas silver method. Twenty-four hours post-injury, APP, RMO14 and NF68 IHC consistently labeled axonal pathologies such as undulated axons, axonal swellings and bulbs in the corpus callosum and the corticospinal tract as described in several published studies [32–35] (data not shown). Argyrophilic axonal degeneration based on Gallyas silver staining became evident 1 week post-injury in the corpus callosum (Fig. 1a), the optic tract and the corticospinal tract (Fig. 1b). Axonal degeneration labeled with Gallyas silver was still present in the corpus callosum (Fig. 1c), corticospinal tract (Fig. 1d) and other white matter tracts 3 months post-injury. These data suggest that the pattern of TAI in nude rates exposed to IA injury is qualitatively similar to the one described for Sprague-Dawley and Wistar rats and, therefore, the nude rat model is suitable for research into hOPC transplantation outcomes in a diffuse TBI background.

### Differentiation of human embryonic stem cells to human oligodendrocyte progenitor cells *in vitro*

As per Hu and colleague’s original description [18, 19], columnar epithelial cells began to appear and organize into rosettes 10 days after induction of differentiation of embryoid bodies with human ESC medium without fibroblast growth factor (FGF)2 (DMEM/F12, Knockout serum replacer, MEM-nonessential amino acid, 0.1 mM β-mercaptoethanol, 4 ng/mL FGF2) for 3 days and then with neural differentiation medium (NDM; DMEM/F12, N2, MEM-nonessential amino acid, 2 ug/mL heparin) for 6 days. Neuroepithelial cells were initially cultured in the presence of 0.1 μM retinoic acid on laminin-coated plates for 4 days as described by Hu and colleagues [18]. The resulting rosette-rich colonies were manually detached and grown into spheres and then continued to be patterned with retinoic acid and SHH for 10 more days. To generate pre-oligodendrocyte progenitors, spheres were passaged by Accutase and cultured in NDM supplemented with B27, SHH (100 ng/mL) and FGF2 (10 ng/mL) for 10 days. For further differentiation into hOPCs, spheres were cultured in GDM (DMEM/F12, N1, B27, MEM-nonessential amino acid, 60 ng/mL T3, 1 μM cAMP, 0.1 μg/mL biotin) supplemented with SHH, PDGF-AA, IGF1 and NT3 for 2 weeks and then dissociated by Accutase and continued to be feed with the same GDM without SHH (day 49). Every 2 or 3 weeks, the spheres were passaged by the same dissociation method using Accutase and cultured in the same GDM with PDGF-AA, IGF1 and NT3. On day 84 after induction of differentiation, spheres were trypsinized into dissociated hOPCs, plated and cultured further as in Materials and methods. On day 98, cells were detached from the plate and resuspended at a high concentration (2.0 × 10^8^ per mL) for transplantation.

**Fig. 2:**
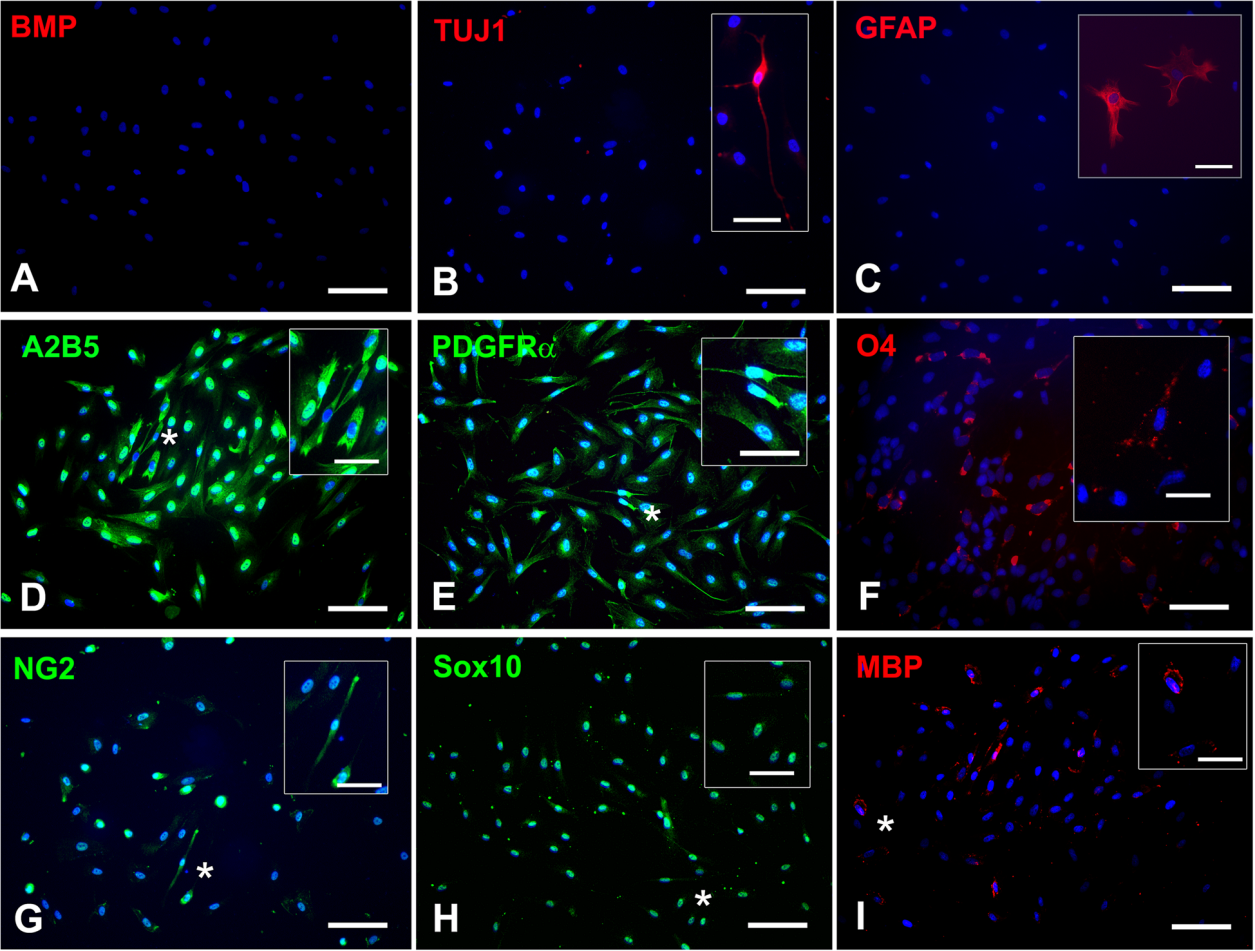
Characterization of human oligodendrocyte progenitor cells used for transplantation 99 days after induction of differentiation of human embryonic stem cells. (**a**) No mesodermal lineage cells were detected (bone morphogenetic protein; BMP) and very little (**b**) neural (type III-tubulin epitope J1; TUJ1) and (**c**) astrocyte (glial fibrillary acidic protein; GFAP) markers were expressed in the oligodendrocyte progenitor cells (OPCs). Most cells (90-95 %) were positive for early and late OPC and pre-oligodendrocyte markers ((**d**) A2B5, (**e**) platelet-derived growth factor receptor (PDGFR)α and (**f**) O4). Fifty percent of cells were (**g**) NG2 positive and (**h**) most cells were positive for the transcriptional factor Sox10. (**i**) About 50 % of cells showed varied expression of the oligodendrocyte marker myelin basic protein (MBP). Insets in (b) and (c) show typical rare neuronal and astrocytic profiles in these human OPC cultures. Inset in (b) is taken from a culture that was double immunostained for NG2 (green) and TUJ1 (red). Insets in (d-i) are magnifications of cells or groups of cells labeled with asterisks to showcase typical cytologies and immunoreactivities, including nuclear (h) and non-nuclear (d,e,f,g,i) localizations. Other than the nuclear localization of the transcription factor Sox10 (H), most immunoreactivities are especially prominent in processes (e,g) or have punctate peripheral localization (f,i). Scale bars = 20 μm

As described in Materials and methods, the cell composition of hOPC transplants was analyzed in a representative sample of cells destined for transplantation with immunocytochemistry for protein markers of various neural cell lineages [36–39] (Fig. 2). Results show that only a small percentage of hOPCs (less than 10 %) expressed the neuronal marker TUJ1, an even smaller percentage (less than 1 %) were positive for astrocytic markers (GFAP), and no bone morphogenetic protein(+) mesodermal-lineage cells were detected (Fig. 2a-c). In contrast, these hOPC samples were enriched for cells expressing oligodendrocyte-lineage markers including A2B5, PDGFRα, O4, NG2, Sox10 and MBP (Fig. 2d-i).

### Survival and migration of transplanted human oligodendrocyte progenitor cells in the rat brain

Human OPCs were transplanted into the deep sensorimotor cortex of IA- and sham-injured rats. Transplanted hOPCs survived very well in the brains of injured and uninjured animals and migrated extensively away from the transplantation site in both groups. We mapped and counted migratory profiles at 6 weeks and 3 months post-transplantation. For the time point of 6 weeks we have data only on IA-injured animals. In the case of 3 months, we have data from both IA-injured and sham animals.

**Fig. 3:**
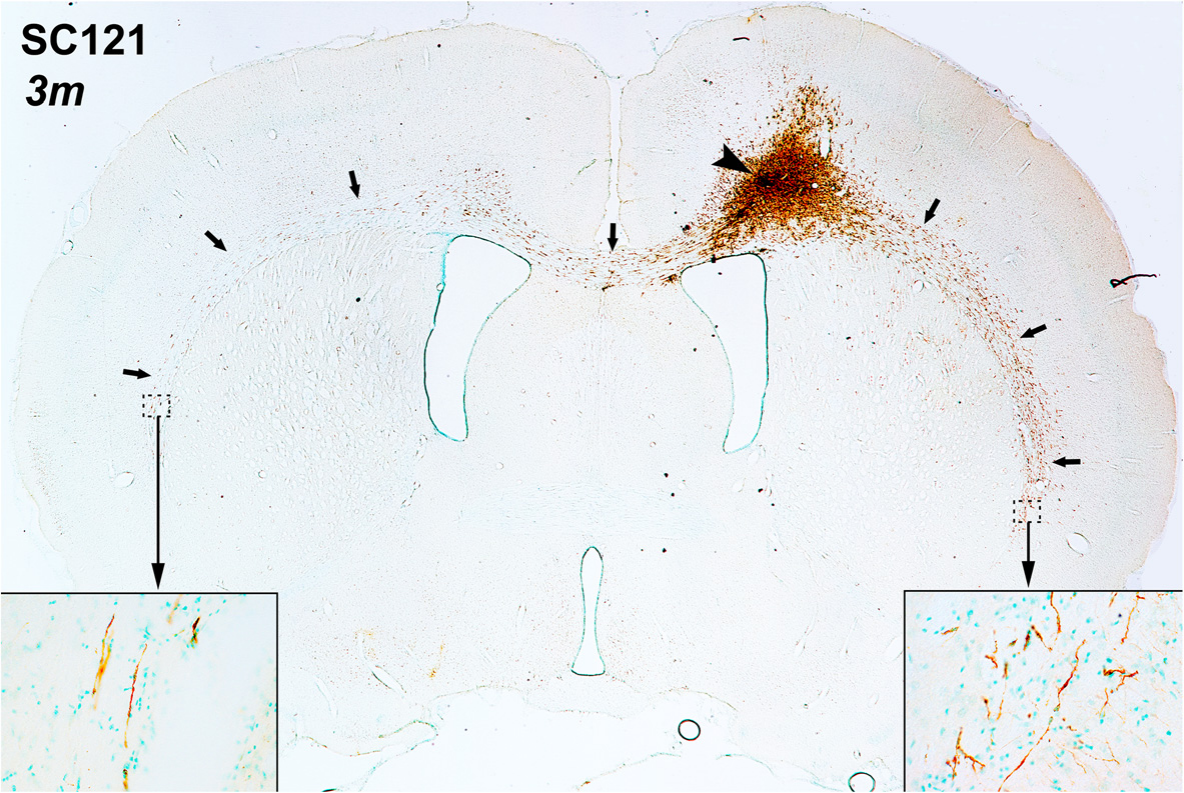
Extensive migration of transplant-derived cells 3 months post-transplantation. This representative section from an impact acceleration-injured rat shows that SC121(+) cells (brown) migrate extensively from the transplantation site (arrowhead) along the corpus callosum on both sides. Methylene green was used as counterstain. Arrows denote human oligodendrocyte progenitor cell distribution. Insets are enlarged from the frames to convey information on cytology

**Fig. 4:**
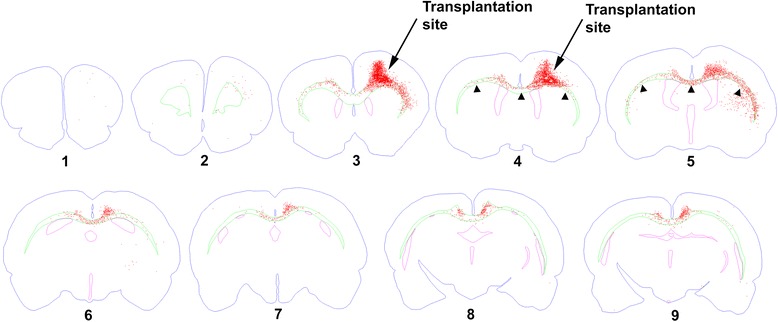
Migration map of human oligodendrocyte progenitor cell transplant-derived cells from a representative case of an impact acceleration-injured rat. Images were acquired and processed from serial coronal sections (40 μm; every 24th) from a case of an impact acceleration-injured rat 3 months post-transplantation using Neurolucida software. Distance between neighboring sections is 0.96 mm. Distance between migrating cells in section 9 and edge of transplantation site (section 4) is 4.8 mm. The maximal migration at 3 months was greater than 5 mm based on the fact that section 9 was not the furthest section containing human cells (other sections are not shown)

At 6 weeks, there was some migration of transplant-derived SC121(+) human cells into the ipsilateral corpus callosum and external capsule and into the contralateral corpus callosum adjacent to cingulate gyrus (Additional file 2: Fig. S2). At 3 months, transplanted cells had migrated much further (Figs. 3 and 4). hOPCs had densely populated the ipsilateral corpus callosum and the entire length of the external capsule and reached further into the corpus callosum and external capsule on the contralateral side; some cells had entered the contralateral neocortex (Fig. 4). In many cases, these cells had migrated 5 mm or more in antero-posterior distance from the transplantation site (Fig. 4). There was little migration into the gray matter, and cortical invasion of hOPCs was limited to layers adjacent to corpus callosum (lower layer 6). In the case of migration into the neostriatum (Fig. 4), hOPCs were localized strictly inside the white matter striae.

**Fig. 5:**
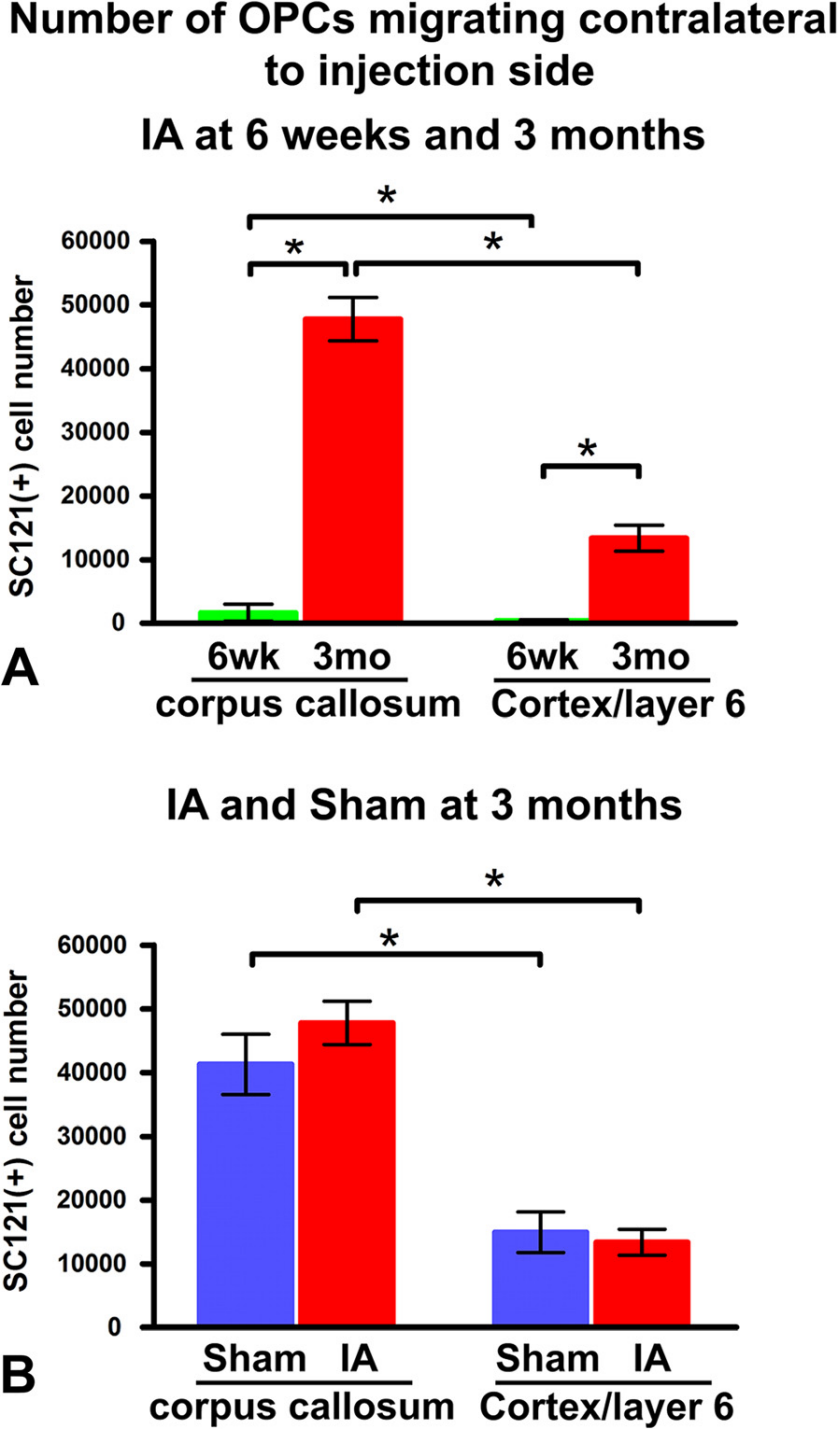
Stereological counts of transplanted human oligodendrocyte progenitor cells migrating into the contralateral hemisphere. **a** Migratory patterns at two time points (6 weeks and 3 months) after transplantation in two brain regions (corpus callosum and deep cortex) in impact acceleration (IA) animals. **b** Migratory tendencies in the same two locations based on experimental history (IA versus sham) at 3 months post-transplantation. In (A), the difference in cell numbers between 6 weeks and 3 months is significant by *t* test (*P < 0.05) in both corpus callosum and deep cortex (layer 6); two-way analysis of variance (ANOVA) shows that there is interaction between time and location (that is, time tends to favor deep cortical over callosal location; *P* < 0.05). In (B), there are no differences in cell numbers between sham and IA-injured subjects in corpus callosum or deep cortex at 3 months post-transplantation. In both groups of subjects, there are more cells in corpus callosum than deep cortex by *t* test (*P* < 0.05). Two-way ANOVA shows that there is no interaction between experimental history and location (that is, injury does not seem to influence the location of cells in one site over the other). OPC, oligodendrocyte progenitor cell

Stereological counts of transplant-derived cells contralateral to the injection side provide a good measure of the migratory potential of transplanted hOPCs. Cell counts in IA-injured animals at 6 weeks and 3 months show massive and progressive migration into the corpus callosum and adjacent cortical layer 6 (Fig. 5). For example, the number of oligodendrocyte-lineage cells in the contralateral corpus callosum is 30 times higher at 3 months compared to 6 weeks (Fig. 5a). Two-way ANOVA examining interaction between time and location shows that time tends to advance the position of cells from the corpus callosum into deep cortical layers (Fig. 5a). Experimental history (IA versus sham) shows no effect on numbers of oligodendrocyte-lineage cells in corpus callosum or cortex at 3 months. There are about three to four times as many oligodendrocyte-lineage cells in corpus callosum compared to cortical layer 6 in both IA-injured and sham animals (Fig. 5b). Two-way ANOVA addressing the interaction between experimental history and location shows no significant effect (that is, lesion does not promote more advanced migration into deep cortical layers).

**Fig. 6:**
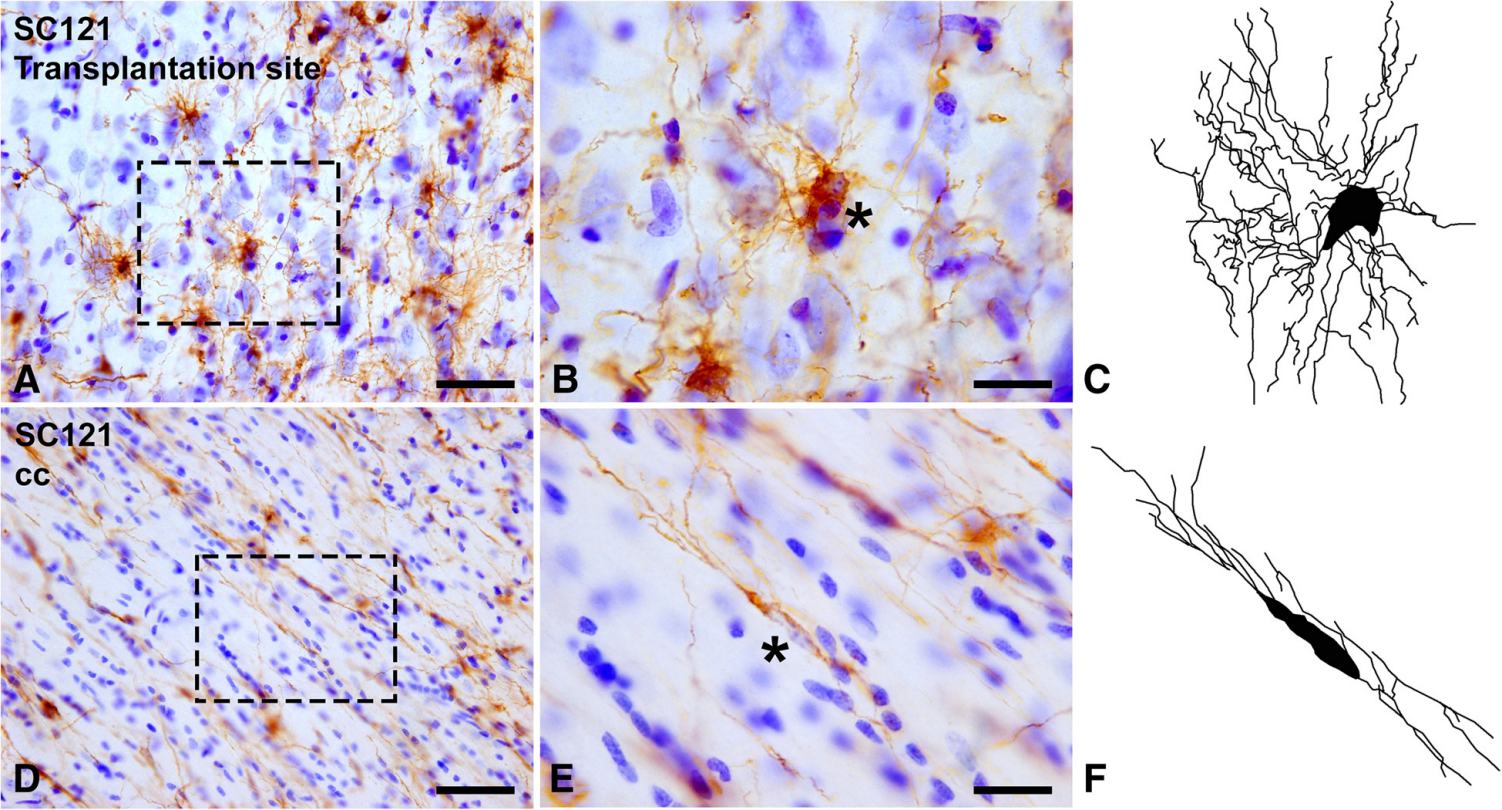
Some cytological features of human oligodendrocyte progenitor transplant-derived cells 3 months post-transplantation. **a-c** Around the transplantation site, a large number of transplant-derived SC121(+) cells (brown) have round features with extensive and radially arrayed processes. **d-f** In the corpus callosum (cc), the majority of SC121(+) cells are spindle shaped with long parallel processes. (b,e) Enlargements of bracketed areas in (a) and (d), respectively. (c,f) Neurolucida tracings of representative cells from (b) and (e) indicated with asterisks. Scale bars: (a,d) = 50 μm; (b,e) = 20 μm

At 3 months post-transplantation, a majority of oligodendrocyte-lineage cells around the transplantation site (the triangular region of Fig. 3) had round perikaryal profiles and multiple radial processes consistent with type I morphology (Fig. 6a-c) [40]. On the other hand, the majority of transplant-derived cells in the corpus callosum were spindle-shaped with parallel processes consistent with type II morphology (Fig. 6d-f) [40]. In the gray matter away from the injection site (ipsilateral or contralateral), cytology was mixed.

**Fig. 7:**
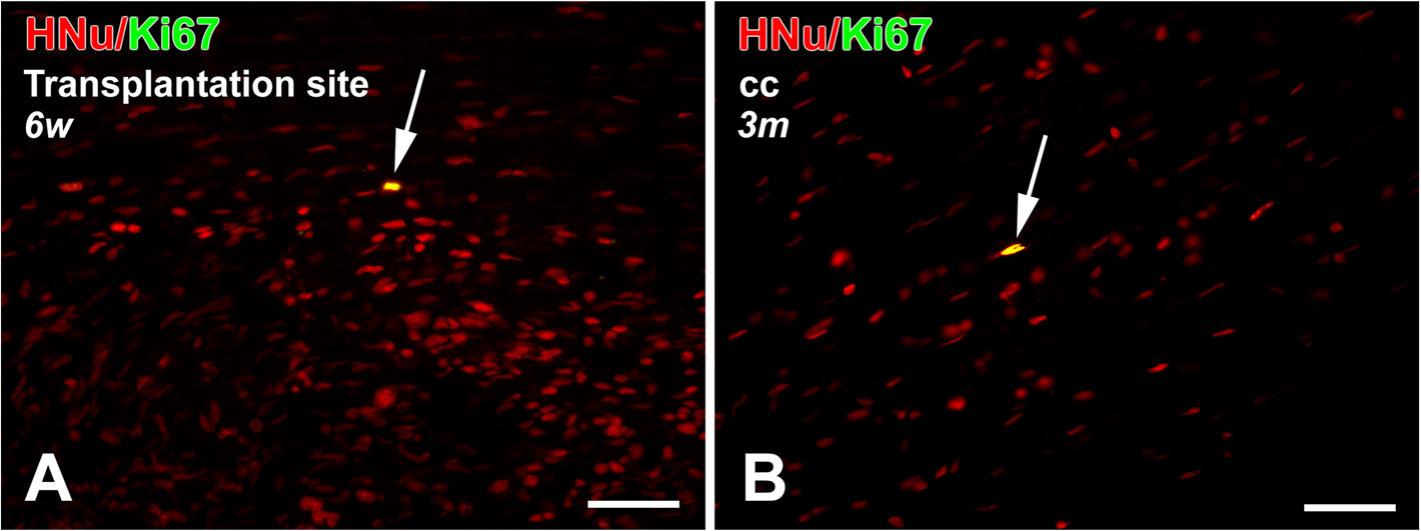
Proliferative activity of human oligodendrocyte progenitor cells at 6 weeks and 3 months post-transplantation. Only rare HNu(+) transplant-derived cells (red) are Ki67(+) cycling cells (green) (arrow, the color turns into yellow because of overlapping with red color from HNu) at the transplantation site at 6 weeks (**a**) or, after migration, in the corpus callosum (cc) at 3 months (**b**). Images are from a representative impact acceleration-injured rat. Scale bars = 50 μm

Very few (less than 1 %) hOPCs at the transplantation site or within the main migratory domains (corpus callosum and deep neocortex) were positive for the mitotic marker-Ki67 at 6 weeks or 3 months, in injured or sham animals. This pattern suggests that, at the time points studied here, surviving cells are not proliferative at the original transplant site or in their migratory paths and destinations (Fig. 7).

### Differentiation of transplanted human oligodendrocyte progenitor cells in the rat brain

**Fig. 8:**
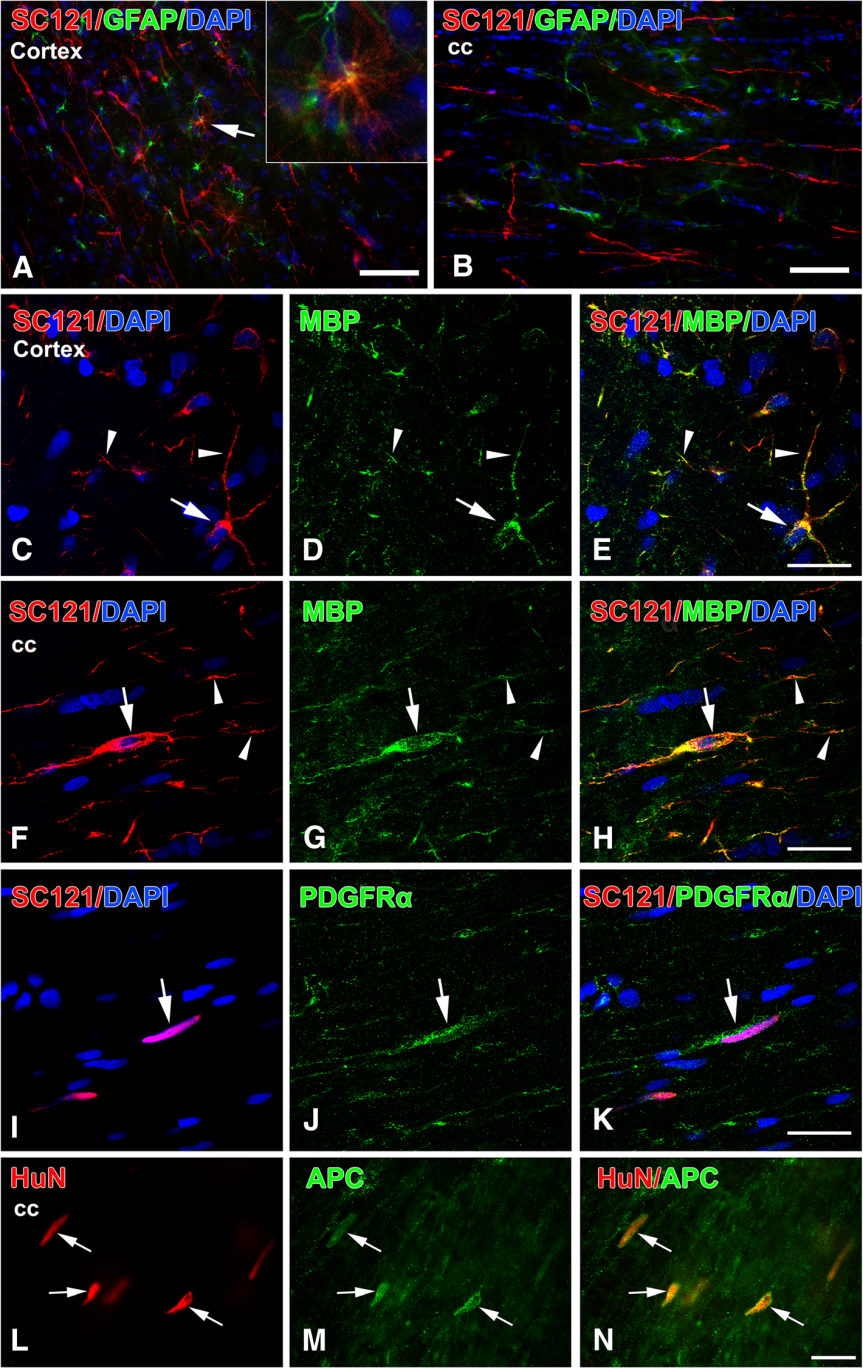
Differentiation of transplanted human oligodendrocyte progenitor cells at 3 months post-transplantation. All images are from representative impact acceleration-injured animals. **a** At 3 months, we found no SC121(+) cells expressing type III-tubulin epitope J1 (TUJ1(+)) neuronal phenotypes, and only rare SC121(+) human oligodendrocyte progenitor cells (hOPCs; red) had differentiated into glial fibrillary acidic protein (GFAP(+)) astrocytes (green) at the transplantation site (arrow). Inset in (a) is a magnification of the astrocytic profile indicated by the arrow in the main panel. **b** In the corpus callosum (cc), no hOPCs (red) are immunoreactive for GFAP (green). **c**-**h** Confocal images to show that various types of cells derived from the hOPC transplant (round in (c) or spindle-shaped in (f), red) become mature myelin basic protein (MBP) (+) oligodendrocytes (green in (d) and (g)); both cell bodies (arrows) and processes (arrowheads) of transplant-derived cells are immunoreactive for MBP. **i-n** Platelet-derived growth factor receptor (PDGFR)α(+) (i-k) and adenomatous polyposis coli protein (APC) (+) (l-n) cell bodies of transplant-derived cells in the corpus callosum. (e,h,k,n) Merged images of panels (c,d), (f,g), (i,j) and (l,m), respectively. Scale bars: (a,b) = 50 μm; (c-n) = 20 μm

At 6 weeks and 3 months post-transplantation, under either IA or sham conditions, no neurons and very few astrocytes were derived from transplanted hOPCs (Fig. 8a,b). The majority of transplanted cells were identified as PDGFRα(+) (Fig. 8i-k) or MBP(+) (Fig. 8c-h) profiles in both the transplantation site and migratory pathways/destinations. MBP immunoreactivity was expressed in both round and spindle-shaped oligodendrocyte profiles derived from the transplant (Fig. 8c-h). At 3 months, most transplant-derived cells around the transplantation site, in corpus callosum, and deep cortical layers were also APC(+) (Fig. 8l-n).

**Fig. 9:**
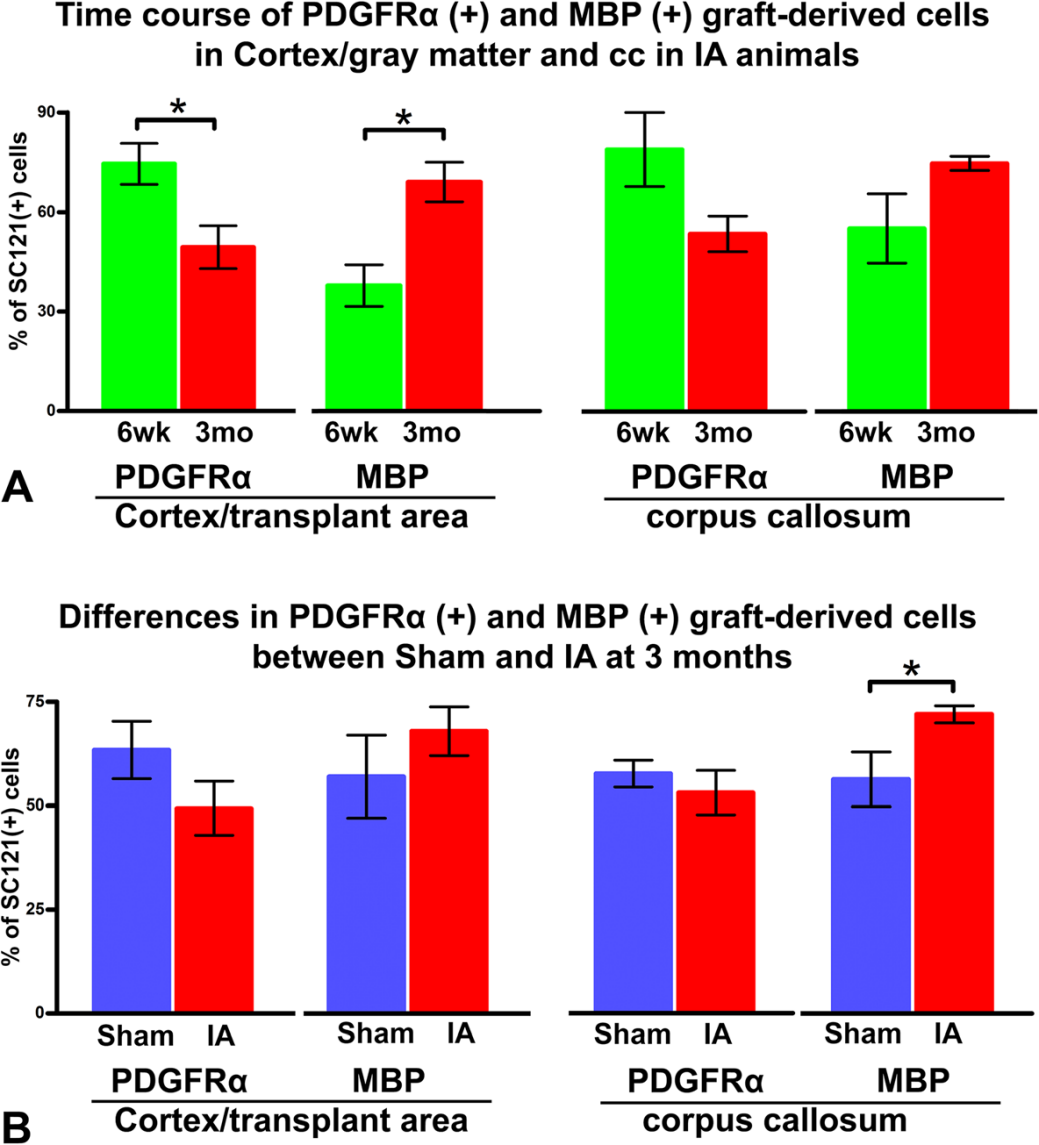
Population sizes of platelet-derived growth factor receptor α- and myelin basic protein-immunoreactive cells derived from human oligodendrocyte progenitor cell transplant. **a** Differentiation patterns at 6 weeks and 3 months after transplantation in two brain regions (corpus callosum versus transplantation area in cortex) in impact acceleration (IA) animals. **b** Differentiation patterns in the same two locations based on experimental history (IA versus sham) at 3 months post-transplantation. In (a), IA rats at 3 months have more myelin basic protein (MBP) (+) and fewer platelet-derived growth factor receptor (PDGFR)α(+) transplant-derived oligodendrocytes than at 6 weeks post-transplantation; counts were performed at the transplantation site. In (b), IA rats at 3 months have more transplant-derived MBP(+) oligodendrocytes compared to sham in the corpus callosum (cc). *P < 0.05

In the area surrounding the transplantation site of IA-injured animals, cell counts of PDGFRα(+) profiles show that 74.6 ± 9 % of graft-derived cells are PDGFRα(+) at 6 weeks post-transplantation; this number is significantly reduced to 49.4 ± 11 % at 3 months. Conversely, the percentage of MBP(+) oligodendrocytes derived from hOPCs is significantly higher at 3 months (67.8 ± 12 %) compared to 6 weeks (37.1 ± 9 %) (Fig. 9a, left). A similar pattern is seen in the corpus callosum (Fig. 9a, right), but trends in this case do not reach statistical significance. In the area surrounding the transplantation site, there are no significant differences in PDGFRα(+) or MBP(+) cell rates between sham and injured animals (Fig. 9b, left). In the corpus callosum, the percentage of MBP(+) cells is significantly higher in injured animals compared to shams (Fig. 9b, right).

**Fig. 10:**
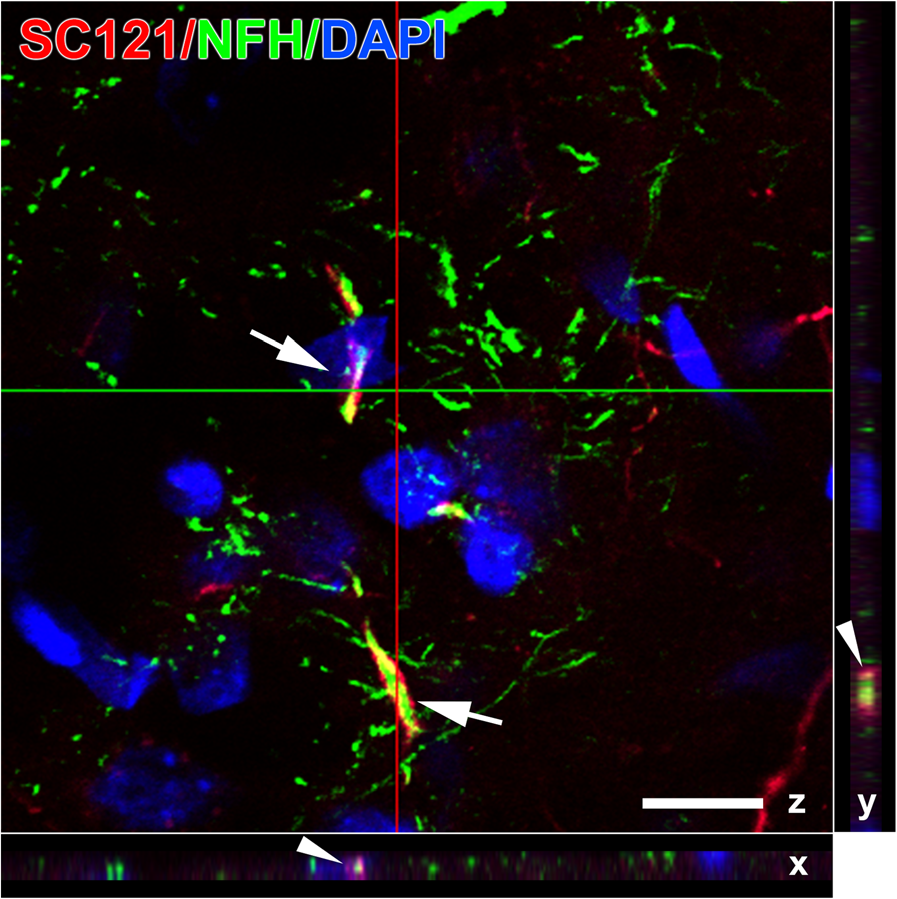
Ensheathment of axons by transplant-derived oligodendrocytes. This three-dimensional reconstructed confocal micrograph depicts SC121(+) process (red) from transplant-derived oligodendrocytes ensheathing neurofilament H(+) axons (NFH, green) in a representative impact acceleration-injured animal at 3 months post-transplantation (arrows on z plane). Ensheathment is confirmed on x and y planes at the corresponding cross-sectional locations (arrow heads). Scale bar = 10 μm

Confocal microscopy with three-dimensional image reconstruction for SC121- and NF-H-immunoreactive structures was used to visualize appositions between SC121(+) processes belonging to transplant-derived cells and NF-H(+) SC121(−) host axons. Reconstructed confocal images demonstrated many ensheathing appositions between processes of transplant-derived oligodendrocytes and host axons (Fig. 10).

**Fig. 11:**
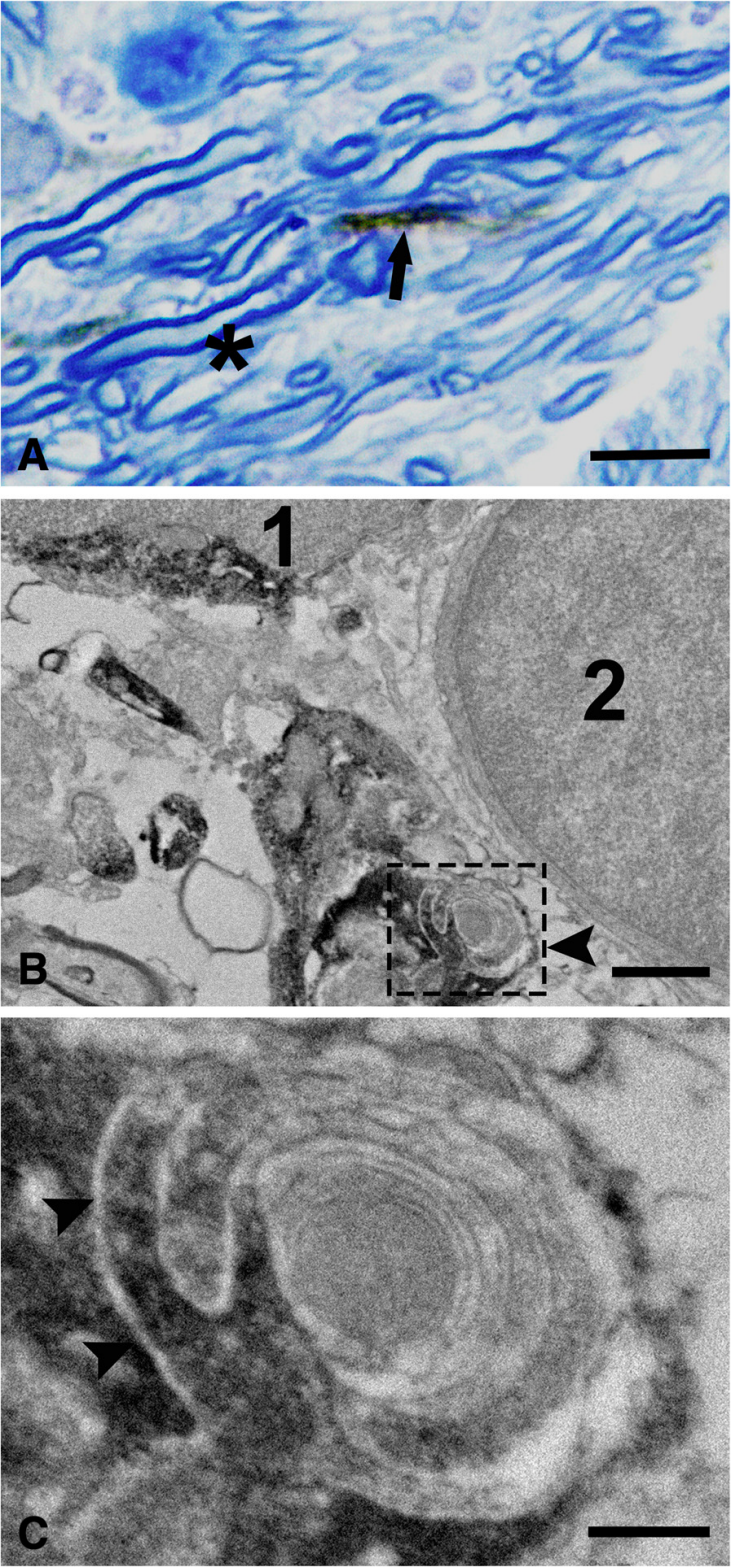
Ensheathing profiles issued by transplant-derived oligodendrocytes as shown by ultrastructural immunohistochemistry. Preparations are from an injured animal 3 month post-transplantation. **a** Companion toluidine blue-stained semi-thin section through the corpus callosum shows the co-localization of SC121(+) (brown) processes with blue myelin sheaths in transverse (arrow) axonal profiles. Co-localization profiles are dark brown. Asterisk shows a group of SC121(−) axons. **b** An SC121(+) process (arrowhead) is shown to ensheath an unlabeled axon. This profile is adjacent to one SC121(+) cell (1) and also one unlabeled cell (2). Cells 1 and 2 have the appearance of oligodendrocytes. **c** A magnification of the framed area in (b) shows detailed ultrastructural features of ensheathment by transplant-derived oligodendrocytes. SC121(+) tongue processes (arrowheads) are wrapped around a myelinated axon. Myelin sheath on the inside appears to be unlabeled. Scale bars: (a) = 5 μm; (b) = 1 μm; (c) = 500 nm

Ultrastructural IHC for the human cytosolic epitope SC121 was used to disclose the involvement of transplant-derived oligodendrocyte processes in the ensheathment of host-derived axons or the formation of myelin. In a pattern similar to the one revealed with confocal microscopy, semi-thin preparations accompanying thin sections showed SC121(+) processes co-localizing with toluidine blue-stained myelin sheaths (Fig. 11a). Using thin sections, we found numerous SC121(+) cytoplasmic projections juxtaposed to or ensheathing unlabeled (host) axons. Ensheathment was featured by complex configurations, including the presence of outer and inner cytoplasmic tongues and close juxtapositions with compact myelin (Fig. 11b,c). It was not possible to ascertain whether compact myelin belonging to the same host axons, as SC121(+) sheaths were continuous with the latter in our preparations because the cytoplasmic human epitope SC121 would not be expected to be present within dense myelin.

## Discussion

Our findings indicate that the IA model of Marmarou can be effectively replicated in the nude rat background. Using the nude rat IA model, hOPC transplants survive well in the deep sensorimotor cortex and behave in a fashion very different from NPs; that is, they migrate massively and show almost exclusive affinity for white matter tracts, especially the corpus callosum and adjacent white matter in deep cortical layers. The progressive migration of transplanted hOPCs is accompanied by progressive maturation into MBP(+) and APC(+) oligodendrocytes that ensheath host axons. Our findings provide further support to the notion that human ESCs and neural stem cells can be coaxed to specific fates that continue to progress to fully differentiated progenies after transplantation into the adult CNS. These progenies behave in a fashion that is strikingly similar to indigenous differentiated neural cells. Given the very low level of proliferation of transplanted cells as early as 6 weeks post-transplantation and their prompt differentiation into mature oligodendrocytes, the possibility of overgrowth and, hence, tumorigenic risk is very low. We postulate that the high numbers of cells present in the contralateral hemisphere by 3 months had started as pre-existing hOPCs in the dense center of the transplant and did not derive from ongoing cell divisions.

The use of human ESCs such as line H9 was based on a number of considerations including: thorough characterization and inexhaustible supply of the parent line [41–44]; great versatility to differentiate to any neural cell type in sufficient quantity for transplantation [18, 43–50]; and well-established methods for *in vitro* manipulation to fate determination prior to transplantation. The choice of human ESCs is based on availability and access considerations, the greater translational value of such cells, and a long experience in our laboratory using human cells as transplants in rodent hosts [21–24, 27, 50–54].

In adulthood, the sources of usable stem cells or neural progenitors in the CNS are limited to a few forebrain niches, and the yield or repair potential of such niches is low. For example, in mouse models of multiple sclerosis, the limited recruitment of endogenous hOPCs into demyelination sites does not suffice for effective remyelination [55]. Therefore, supplementation of such limited stem cell pools with exogenous progenitors is a reasonable first step for a cellular therapy. Besides providing sources of fully differentiated nerve cells competent to replenish lost cells, transplanted progenitors also release neuroprotective molecules [27, 56] and, importantly, may induce endogenous stem cells/progenitor cells to proliferate and differentiate as auxiliary niches, thereby improving the efficacy of self-repair mechanisms [52].

### Generation of human oligodendrocyte progenitor cells from human embryonic stem cells

In the body of *in vivo* studies reported here, we used hOPCs that were prepared from human ESC line H9 following the methods described by Hu and colleagues with minor modifications [19, 45, 57]. Our experience with culturing and differentiating H9 cells and then characterizing the derived hOPCs *in vitro* is very similar to the original description of Hu and colleagues; hOPCs derived in this manner express PDGFRα, NG2, O4, and Sox10, a pattern consistent with a classical hOPC identity [36]. Methodological issues concerning hOPC derivation are very important, because the use of highly concentrated hOPCs for grafting is key for achieving the desired outcomes (that is, myelination of host axons) within a limited time period.

Differentiation of human ESCs into hOPCs is a longer and more arduous process than the one leading to neuronal progenitors. In work reported here we initially used two methods [18, 58] for deriving and characterizing hOPCs prior to transplantation. In our hands, the method of Hu and colleagues appeared to be more successful in generating viable transplants, although this observation was not confirmed in a systematic fashion. Major differences between the two methods are: trophic factors used; extracellular matrix used; enzyme used to passage the cells; length of time in three-dimensional culture; and timing of hOPC harvest. In previous published work, the method of Nistor and colleagues generated hOPCs with good viability after transplantation [58–62], but the transplantation site of these authors (spinal cord) was different from ours (neocortex). Interestingly, the same team of investigators reported that their hOPCs did not survive past 2 weeks after transplantation in animal models of multiple sclerosis, but these mice were immunocompetent C57B6 mice [63]. Our *in vivo* outcomes using hOPCs prepared as per Hu and colleagues are consistent with the ones reported by that team on shiverer mice [19].

### Issues related to *in vivo* differentiation of human oligodendrocyte progenitor cell transplants

Two important trends in hOPC maturation were the progressive phenotypic differentiation in the transplantation area and the higher rate of maturation of hOPCs in the corpus callosum of injured subjects. With respect to the former, we have observations only from IA-injured animals, but we speculate that there is a similar differentiation trend in control (sham) animals. We also postulate that differentiation trends in the corpus callosum are not too dissimilar to those in the cortex around the transplantation site and that differences in significance may be caused by the fact that hOPC maturation may be earlier in the white matter [64] compared to grey matter. Regarding the latter, it would appear that injury may contribute to hOPC differentiation; the difference between corpus callosum and cortex around the transplantation site may be due to the fact that cortex is not a primary site of injury in the IA model that preferentially affects white matter tracts. We have previously reported that the injured spinal cord niche influences both the proliferation and differentiation of human CNS stem cells propagated as neurospheres [65].

The role of injured or otherwise pathological environment as a niche for differentiation deserves further commentary. Environments associated with acquired neurological injury such as stroke and spinal cord injury have been shown to promote endogenous stem cell differentiation [9, 66, 67] and this effect may be mediated, in part, by trophic and cytokine signals such as stem cell factor [68], stromal cell-derived factor 1α [69], FGF-2 [70], vascular endothelial growth factor [71], ciliary neurotrophic factor, and CXC chemokine receptor 4 [72]. Some of these factors are known to act as tropic cues for migration or to specify the definitive phenotype of endogenous or exogenous stem cells [68, 73–79].

The invasion of migrated hOPCs and their differentiated progenies into deep cortical layers matches native cortical myelination patterns; these patterns show an overall denser myelination in lower cortical layers and variable myelination in superficial layers. It is also interesting that, at least up to 3 months post-transplantation, the differentiated progeny of transplanted hOPCs does not advance to more superficial layers. This distribution is identical to that of native mature oligodendrocytes; in contrast to hOPCs that are radially spread across cortical layers, oligodendrocytes favor deep layers and follow the deep-to-superficial-layer myelin gradient [80].

The presumed intent of OPC transplants as cell therapies is the replenishment of damaged or destroyed myelin sheaths. Although molecular myelin markers such as MBP may be telling of the myelin-forming potential of OPCs and their progenies, they do not directly show that MBP(+) cells are forming myelin. The three-dimensional reconstruction of confocal images indicates a close, ensheathment-like, apposition of host axons with processes of transplant-derived oligodendrocytes, but the presence of structurally mature myelin can only be ascertained ultrastructurally. Using ultrastructural IHC or electron microscopy combined with histochemistry, previous studies have demonstrated the ability of specific progenies of neural stem cells or OPCs to ensheath [21] or increase the thickness of abnormal myelin sheaths in hosts [19, 26, 59, 61, 81, 82], but labeling of exogenously derived myelin is technically difficult. In the present study, ultrastructural IHC divulges that transplant-derived cells ensheath host axons in intimate proximity to myelin sheaths, but the host-versus-transplant identity of the myelin itself is difficult to ascertain. The human cell marker SC121 is a cytosolic marker and, therefore, it is found in oligodendrocyte cytoplasmic projections and ensheathing tongues but not in the membranous myelin sheath itself. Unstained preparations that were used here have not been particularly useful, primarily because of this reason. Furthermore, existing myelin antibodies cannot resolve between human and rodent myelin and this problem limits their usefulness in confocal microscopy or ultrastructural IHC.

Ultimately, the proof of concept that remyelination or myelin remodeling by OPC transplants can be beneficial in TAI/DAI will depend on the demonstration that such exogenous OPCs afford functional benefits. In the case of IA-injured rodents, such benefits can be sought out in a number of behavioral domains. One approach is assessment of motor control, which depends on the intactness of the corticospinal tract. Experiments addressing functional repair with exogenous OPCs have been successfully performed in models of spinal cord injury [10, 11] and demyelination [82–85]. In TBI models, motor recovery may occur within the first month after injury in the absence of any therapy, making the assessment of the efficacy of cell therapies more challenging. In such models, the assessment of efficacy or human neural cell therapies may be more straightforward using tasks of cognition or anxious/emotional disposition, faculties that become chronically impaired in rodent TBI. Chronicity of deficits is important because these human cells may take months to proliferate, migrate and terminally differentiate [15].

### Stem cell transplantation as experimental therapy for traumatic brain injury

Some success in models of ischemic brain injury [9] has encouraged the use of stem cell/NP transplantation in models of focal TBI [10, 86]. However, because of the complexity of TBI and its animal models, there is a need to identify specific repair targets based on key pathological mechanisms. Such repair tasks include replacing dead neurons, supporting injured neurons, and protecting axons or assisting with axonal repair/regeneration. The problem of neuronal injury/death is encountered both in focal injury [87, 88] and in the course of TAI [89, 90]. Neuronal cell death in focal TBI is acute and has necrotic components, whereas in TAI/DAI it is slow with apoptotic features and may be associated with retrograde and trans-synaptic effects [8, 89, 91]. Although axonal repair/remyelination as a therapeutic target separate from neuronal regeneration is best established in spinal cord injury [11], there is evidence that demyelination may contribute to degeneration of axons in TAI [12, 13]. Therefore, contributing exogenous hOPCs in the case of TAI may assist in remyelination and prevent axonal degeneration and disconnection within brain circuits.

There is very little published work on stem cell-based therapies for models of TAI/DAI. However, the field of TBI and more specifically TAI/DAI can borrow from spinal cord injury that invariably involves trauma in long tracts [59, 60, 92–96]. Experimental cell therapies in animal models of spinal cord injury have utilized various stem cell preparations including neurospheres and OPCs [59, 60, 96, 97], and there are several ongoing clinical trials using neural stem cells [98, 99]. In one report, OPCs were found to remyelinate and restore locomotion after contusional spinal cord injury in rodents [59], but functional recovery reported in this study occurred within 12 days of transplant, a time point that is too early with respect to migration and terminal differentiation of OPCs. In contrast to spinal cord injury, where long tracts course in relatively circumscribed areas, DAI involves disparate white matter tracts [100, 101] and it would be difficult to transplant cells into all these sites. Therefore, transplantation route (systemic, ventricular, and parenchymal) and location of transplant (in the case of parenchymal delivery) are critical. The choice of transplantation site may be based on factors such as concentration of axonal pathology or sites of injury responsible for critical symptoms. The choice of transplantation into deep sensorimotor cortex in the present study was based on the expectation that this site would provide oligodendrocytes for both the corpus callosum and the corticospinal tract, which are affected in the IA injury [16, 35]. Our findings indicate that there was little, if any, invasion of cells into the internal capsule by 3 months, but the extensive migration of OPCs via the corpus callosum and descending tracts and their remarkable differentiation into mature oligodendrocytes predicts a broader remyelination potential with longer survival times. Of course, transplantation sites can also be optimized to the desired functional outcome or involve multiple locations as we have shown in models of motor neuron disease [23, 52, 53].

## Conclusions

In conclusion, the findings in this study support the idea that hOPCs can serve as a competent source of mature oligodendrocytes that ensheath CNS axons after TAI, and provide proof of concept that regenerative strategies targeting myelin remodeling can be further considered in TBI models in the future. In addition, we demonstrate that the nude rat is a suitable animal model for studying human cell transplants in neurotrauma. In view of the fact that stem cell therapies are being progressively introduced in clinical trials of neurodegenerative and traumatic diseases of the CNS [11, 98, 102–108], these timely results should encourage further translational work targeting the problem of axonal degeneration in the context of DAI and, specifically, interventions designed to regenerate or remodel the myelin sheath.

## Additional files

Additional file 1: Fig. S1. A schematic illustration (A) of the method used to prepare hOPCs for transplantation and representative cultures and cells derived (B). Method sketched in A was based on Hu and colleagues [18] with minor modifications in the final stage before transplantation (day 84–99). Panel B shows representative morphologies of hOPCs on days 3, 7, 12, 17, 23, 27, 41, 90 and 97 that roughly correspond to milestones in A. Human ESC H9 colonies were detached by dispase on day 1 to prepare embryoid bodies (EBs) that were subsequently cultured for 3 days in hES medium without FGF (B, *Day 3*) and then for 3 days in NDM. Embryoid bodies (B, *Day 7*) were then plated on laminin-coated plates and cultured with NDM for 3 days and NDM with RA for another 5 days (B, *Day 12*). On day 15, colonies were manually detached and cultured as spheres (B, *Day 17*) for 10 days in NDM with RA, B27 and SHH. On day 24, big spheres (B, *Day 23*) were dissociated by Accutase (ACT) into small spheres (B, *Day 27*) and cultured with NDM containing B27, SHH and FGF for 10 days. On day 35, medium was switched to GDM with SHH, PDGF, IGF and NT3 and cultured for 2 weeks (B, *Day 41*). On days 49 and 70, cells were passaged with Accutase treatment and treated with GDM with PDGF-AA, IGF1 and NT3 for the remaining of the protocol. On day 84, spheres were trypsinized and plated on p-L-ornithine and laminin-coated plates and coverslips and cultured for 2 weeks in the same medium for transplantation or immunocytochemistry, respectively. On day 99, cells were trypsinized with TrypLE, counted, resuspended in high concentration and used for transplantation (* in A). Scale bars in B: Day 12, 500 μm, Day 3 and 17; 200 μm, all others; 100 μm. Additional file 2: Fig. S2. Migration of transplant-derived cells at 6 weeks and 3 months post-transplantation. Photographs are taken through the corpus callosum on the side contralateral to transplantation from representative animals. Panel A shows a few scattered SC121(+) profiles at 6 weeks. At 3 months post-transplantation (B), there are numerous SC121(+) cells that had migrated from the transplantation site. Insets are enlargements of framed areas in main panels. Scale bars: 100 μm.

## Abbreviations

ANOVA: analysis of variance
APC: adenomatous polyposis coli protein
APP: amyloid precursor protein
CNS: central nervous system
DAI: diffuse axonal injury
DMEM: Dulbecco's modified Eagle's medium
ESC: embryonic stem cell
FGF: fibroblast growth factor
GDM: glial differentiation medium
GFAP: glial fibrillary acidic protein
hOPC: human oligodendrocyte progenitor cell
IA: impact acceleration
IGF: insulin-like growth factor
IHC: immunohistochemistry
MBP: myelin basic protein
NDM: neural differentiation medium
NP: neural precursor
NY: neurotrophin
OPC: oligodendrocyte progenitor cell
PDGF: platelet-derived growth factor
PDGFR: platelet-derived growth factor receptor
SHH: sonic hedgehog
TAI: traumatic axonal injury
TBI: traumatic brain injury
TUJ1: type III-tubulin epitope J1
