# Supplementary figures and images for: Transplantation of human oligodendrocyte progenitor cells in an animal model of diffuse traumatic axonal injury: survival and differentiation
Source: Stem Cell Res Ther. 2015 May 14;6(1):93. doi: 10.1186/s13287-015-0087-0 (PMC4453242; doi:10.1186/s13287-015-0087-0)

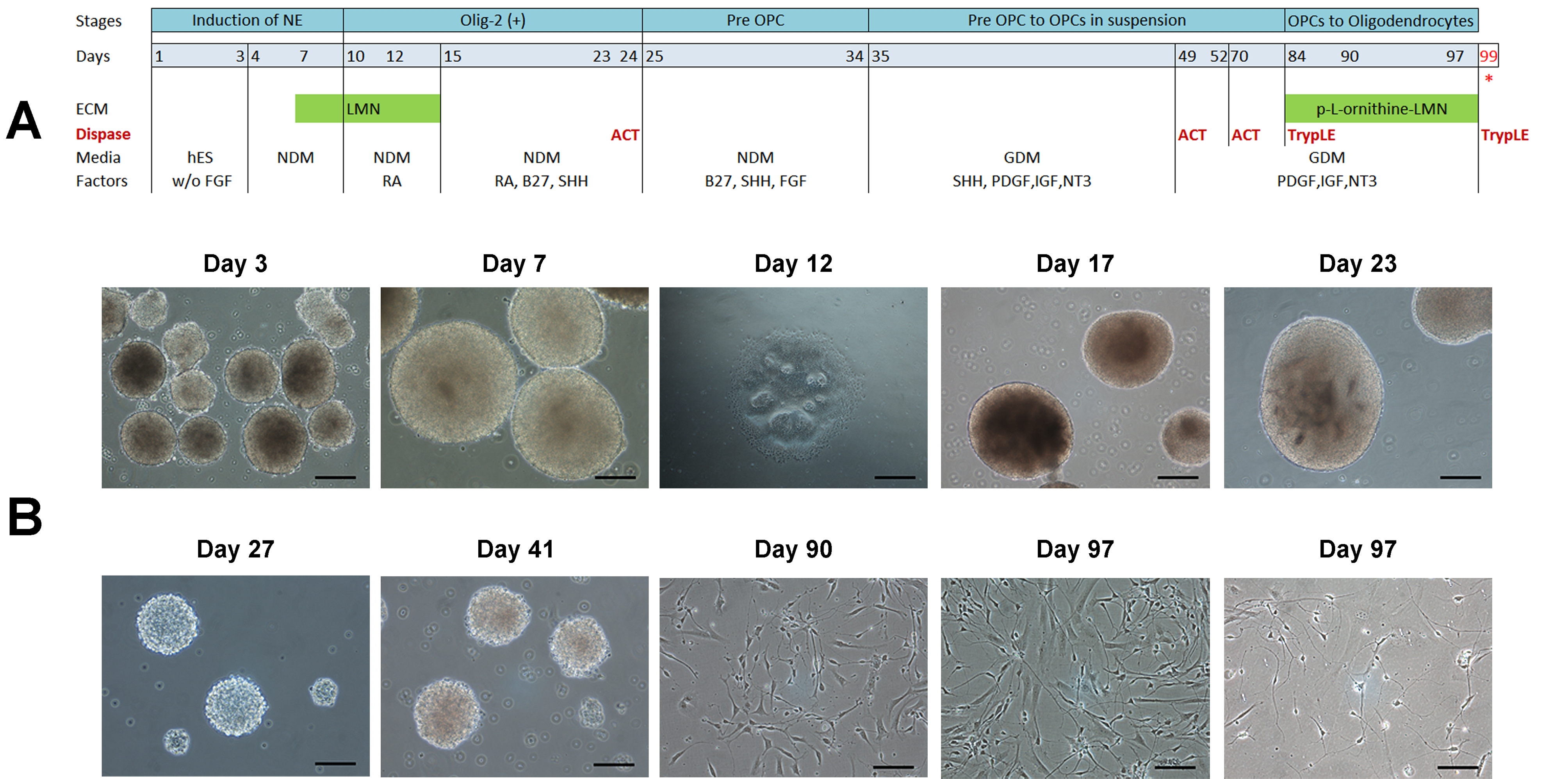

Supplement: Additional file 1: Fig. S1. — A schematic illustration (A) of the method used to prepare hOPCs for transplantation and representative cultures and cells derived (B). Method sketched in A was based on Hu and colleagues [18] with minor modifications in the final stage before transplantation (day 84–99). Panel B shows representative morphologies of hOPCs on days 3, 7, 12, 17, 23, 27, 41, 90 and 97 that roughly correspond to milestones in A. Human ESC H9 colonies were detached by dispase on day 1 to prepare embryoid bodies (EBs) that were subsequently cultured for 3 days in hES medium without FGF (B, Day 3) and then for 3 days in NDM. Embryoid bodies (B, Day 7) were then plated on laminin-coated plates and cultured with NDM for 3 days and NDM with RA for another 5 days (B, Day 12). On day 15, colonies were manually detached and cultured as spheres (B, Day 17) for 10 days in NDM with RA, B27 and SHH. On day 24, big spheres (B, Day 23) were dissociated by Accutase (ACT) into small spheres (B, Day 27) and cultured with NDM containing B27, SHH and FGF for 10 days. On day 35, medium was switched to GDM with SHH, PDGF, IGF and NT3 and cultured for 2 weeks (B, Day 41). On days 49 and 70, cells were passaged with Accutase treatment and treated with GDM with PDGF-AA, IGF1 and NT3 for the remaining of the protocol. On day 84, spheres were trypsinized and plated on p-L-ornithine and laminin-coated plates and coverslips and cultured for 2 weeks in the same medium for transplantation or immunocytochemistry, respectively. On day 99, cells were trypsinized with TrypLE, counted, resuspended in high concentration and used for transplantation (* in A). Scale bars in B: Day 12, 500 μm, Day 3 and 17; 200 μm, all others; 100 μm. [file 13287_2015_87_MOESM1_ESM.tiff]

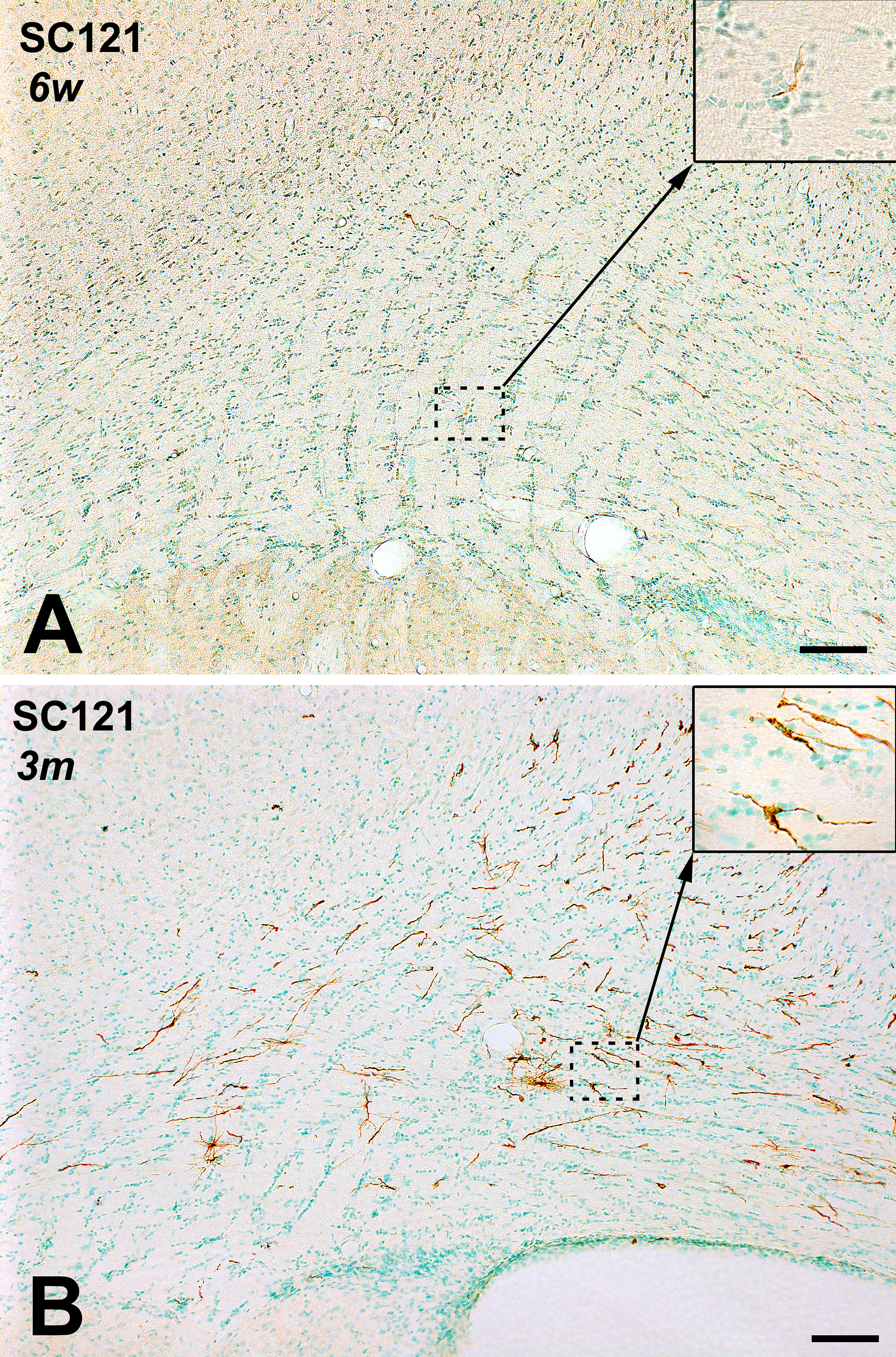

Supplement: Additional file 2: Fig. S2. — Migration of transplant-derived cells at 6 weeks and 3 months post-transplantation. Photographs are taken through the corpus callosum on the side contralateral to transplantation from representative animals. Panel A shows a few scattered SC121(+) profiles at 6 weeks. At 3 months post-transplantation (B), there are numerous SC121(+) cells that had migrated from the transplantation site. Insets are enlargements of framed areas in main panels. Scale bars: 100 μm. [file 13287_2015_87_MOESM2_ESM.tiff]
